# Male Urethral Stricture in Patients with Metabolic Syndrome

**DOI:** 10.5152/tud.2023.22129

**Published:** 2023-03-01

**Authors:** Ahmet Asfuroglu, Melih Balci, Burak Koseoglu, Cagdas Senel, Ali Yasin Ozercan, Ibrahim Can Aykanat, Mehmet Yildizhan, Ozer Guzel, Yilmaz Aslan, Altug Tuncel

**Affiliations:** 1 Department of Urology, Ankara City Hospital, Ankara, Turkey; 2 Department of Urology, Memorial Bahçelievler Hospital, Istanbul, Turkey; 3 Department of Urology, Balıkesir University Faculty of Medicine, Balıkesir, Turkey; 4 Department of Urology, Koç University Faculty of Medicine, Istanbul, Turkey

**Keywords:** Infection, metabolic syndrome, recurrence, urethral stricture

## Abstract

**Objective:**

Urethral stricture is characterized by fibrosis that decreases urine flow. Metabolic syndrome is a complex disorder that causes fibrosis in many organs. This study aimed to evaluate the relationship between metabolic syndrome and appearance of urethral stricture and effects of metabolic syndrome on the recurrence of urethral stricture in patients with primary urethral stricture who underwent direct visual internal urethrotomy.

**Materials and methods:**

One hundred thirty-two male patients who underwent direct visual internal urethrotomy between 2014 and 2021 because of primary urethral stricture were included. Location, length, and type of urethral stricture, time from diagnosis to surgery, postoperative follow-up, time from surgery to recurrence, and postoperative follow-up duration with a urethral catheter were retrospectively analyzed and association with metabolic syndrome was evaluated.

**Results:**

The mean age was 50.48 ± 17.94 years. Recurrence was found in 34.1% and metabolic syndrome in 27.3%. Postoperative follow-up duration was significantly longer in patients with recurrence than in those without (*P* = .033). There was no statistically significant difference in terms of metabolic syndrome and postoperative urethral catheterization between patients with and without recurrence (*P* = .126, *P* = .714, respectively). Postoperative clean intermittent self-catheterization use was found to be statistically higher in patients with recurrence than in patients without recurrence (*P* = .018). Postoperative urinary tract infection rate was found to be significantly higher in patients with metabolic syndrome compared to patients without metabolic ­syndrome (*P* = .001).

**Conclusion:**

Metabolic syndrome was not associated with recurrence. However, postoperative urinary tract infections were more common in patients with metabolic syndrome than in patients without. Clean intermittent self-catheterization used postoperatively may increase the risk of stricture.

**Main Points**

- Postoperative urinary tract infections were more common in patients with metabolic syndrome.
- There was no relationship between recurrence of urethral stricture and metabolic syndrome.
- Clean intermittent self-catheterization used postoperatively was found to increase the possibility of stricture.

## Introduction

Urethral stricture, which is characterized by fibrosis in the subepithelial tissue and corpus spongiosum, is a recurrent disease that narrows the lumen and reduces urine flow, with lower urinary tract symptoms and decreased maximum urinary flow rate (*Q*_max_).^1^ Untreated urethral stricture can lead to life-threatening complications including urinary tract infection (UTI), acute urinary retention, hydronephrosis, and periurethral abscess.^2,3^ The prevalence of urethral stricture is 0.2%-0.6%.^2^ Urethral stricture can be treated with endoscopic or open (conventional) surgeries.^2^ Endoscopic surgeries are direct visual internal urethrotomy (DVIU) that can be performed with cold knife or laser ablation and urethral dilatation.^2^

The most common cause of urethral stricture is iatrogenic, accounting for 45% of cases; the other factors are external trauma, infection, lichen sclerosis, and idiopathic.^4,5^ The connective tissue of the normal urethra and corpus spongiosum consists of type 1 and type 3 collagen. In urethral fibrosis, the proportion of type 1 to type 3 collagen alters, which leads to urethral stricture.^1^ Metabolic syndrome (MetS) is a multivariate and complex disorder characterized by insulin resistance, dyslipidemia, central obesity, and endothelial dysfunction.^6^ Hormones and cytokines released from excess adipose tissue can cause nitric oxide deficiency, which damages the vascular endothelial mechanism, and MetS.^7,8^ Several urologic disorders, including male infertility, erectile dysfunction, benign prostatic hyperplasia, prostate cancer, urinary incontinence, and urolithiasis, are associated with MetS, with a predisposing role.^6^ In addition, MetS may lead to fibrotic processes in many organs such as the liver and heart.^9,10^ Based on this knowledge, it was predicted that MetS, which can cause fibrotic events in tissues, might cause the formation or recurrence of urethral stricture developing on a fibrotic background, and the effect of MetS on urethral stricture and recurrence was investigated in patients with urethral stricture for the first time (primary urethral stricture) who underwent DVIU with a cold knife.

## Materials and Methods

### Patients’ Selection

In this study, 153 male patients who underwent DVIU between 2014 and 2021 with primary urethral stricture were included retrospectively. Since there was no similar study, when the alpha error was calculated to be 0.05 and the effect size to be 0.6 in the G-power analysis we conducted within our own study, 45 patients in each group were required for 0.80 power. The patients with a urethral stricture longer than 2 cm were not included. In the perioperative evaluation of all patients, urethrography was performed by inflating the catheter balloon 3 cc with a 14-F catheter in the fossa navicularis. Patients aged over 18 years and diagnosed with primary urethral stricture were included in this study. Patients who were admitted with a secondary intervention (DVIU for the second time) plan were excluded from the study. Among the patients included in the study, 11 patients with a history of hydronephrosis due to urethral stricture and 10 patients with a history of cystostomy catheter due to acute urinary retention were excluded. The 132 patients included in the study were divided into 2 groups according to the presence of recurrence during their follow-up with uroflowmetry and postvoiding residual volume (PVR). Group 1 consisted of 45 patients with recurrence, and group 2 consisted of 87 patients without recurrence. In addition, patients were divided into 2 groups in line with the recommendations in the American Urology Guidelines as 0-3 days and 4 and more days according to the duration of the Foley urethral catheter insertion.^11^

The parameters of uroflowmetry including *Q*_max_ (mL/s), mean urinary flow rate (*Q*_ave_) (mL/s), voiding volume (VV) (mL), and PVR (mL) in the preoperative period, in the first month postoperatively, every 1-6 months according to the treatment of patients, and at the time of recurrence in patients in whom urethral stricture recurred, were evaluated from the patient files. Preoperative and postoperative history of UTI, time from diagnosis to surgery, postoperative follow-up duration, and in patients with postoperative recurrence time from surgery to recurrence were recorded. The location, length (cm), and type (as pinpoint or annular) of urethral stricture were recorded from the patients' operative records. The postoperative follow-up duration of the patients with a urethral catheter was noted. We decided to clean intermittent self-catheterization (CISC) based on the length and severity of the urethral stricture. We recommend CISC if the patient can also use it in long and severe strictures. The use of CISC catheters was recorded in patients who were able to apply.

In our study, urethral stricture in uroflowmetry with voiding pattern and *Q*_max_ of 10 or less were considered as recurrence. Urethral stricture length, *Q*_max_, *Q*_ave_, difference of *Q*_max_ (postoperative *Q*_max_ – *Q*_max_ in recurrence), VV, and PVR were evaluated in patients with recurrence. Similar groups according to the presence of recurrence of urethral stricture were formed with the evaluation of age, PVR, *Q*_max_, and urethral stricture length in patients, and comparison was evaluated between these groups.

### Metabolic Syndrome Analysis and Clinical Data Selection

The patient records of the urology clinic where the patients underwent surgery and were followed up and the records of the national patient database were analyzed retrospectively. The diagnosis of MetS was made according to the most recent consensus report of the National Cholesterol Education Program’s Third Adult Treatment Panel III (NCEP ATP III) recommendations.^12^ According to NCEP ATP III, MetS is the occurrence of at least 3 of 5 risk factors including abdominal obesity (waist circumference >102 cm), hypertension (blood pressure >130/85 mm Hg or being on antihypertensive medication), hyperglycemia (fasting blood glucose >110 mg/dL), hypertriglyceridemia (serum triglyceride levels ≥150 mg/dL), and reduced levels of high-density lipoprotein-cholesterol (HDL-C) (<40 mg/dL).^12^ Patients were divided into 2 groups (group A and group B) according to the presence of MetS. Group A consisted of 36 patients with MetS, and group B consisted of 96 patients without MetS. Blood pressure (mm Hg), body mass index (kg/m^2^), waist circumference (cm), and biochemical analyses including serum glucose, triglyceride, and HDL-C (all as mg/dL) levels of the patients were noted from the records. In addition, medical history and smoking history were also noted.

### Surgical Technique

Under general or major regional anesthesia with a 21-F urethrotome, visual urethrotomy was performed by cutting the stricture with a cold knife at the 12 o’clock position according to the technique described by Sachse.^13^ Postoperatively, according to the width of the urethral lumen or surgeon’s preference, a 12-22-F urethral catheter was indwelled and left for 1-14 days, in concordance with the literature. In the postoperative period, in 26 patients, 14-F CISC was used, and they were recommended to be used twice per week for 3 months.

### Statistical Analysis

The IBM SPSS Statistics version 22.0 (IBM SPSS Corp.; Armonk, NY, USA) for Windows was used for the statistical analyses. For descriptive statistics, categorical variables are expressed as absolute numbers and percentages, and continuous variables are expressed as mean ± SD. The normal distribution of continuous variables was tested using histograms and the Kolmogorov –Smirnov test. The independent samples *t*-test was used for independent variables and the paired *t*-test for dependent variables for normally distributed variables. The chi-square test was performed in the analysis of categorical variables. Kaplan –Meier survival analysis was used in the recurrence-free survival analysis of the patients. For matched pair analysis, logistic regression analysis and propensity score matching were performed. A *P*-value of <.05 was considered statistically significant.

This study was conducted in accordance with the amended Declaration of Helsinki, and all procedures performed in studies involving human participants were prepared in accordance with the Ethics Committee of Ankara City Hospital (ethics committee approval date: 26/05/2021; number: E1/1787/2021). Informed consent was obtained from all patients.

## Results

The mean age of the patients was 50.48 ± 17.94 (18-86) years. When the etiology of urethral stricture in the patients was evaluated, 87.9% were iatrogenic (endoscopic surgery: n = 86; urethral catheterization: n = 30), 6.8% were external trauma, and 5.3% were UTIs. The median follow-up duration was 28.0 (4.0-81.0) months. Of the patients, 60.6% had a smoking history, 22.7% had type 2 diabetes mellitus, 61.4% had a history of essential hypertension that did or did not require antihypertensive medication, and 32.6% had coronary artery disease. The comparison of demographics and perioperative data of the groups is shown in Table 1.

Metabolic syndrome was present in 27.3% of the patients. The laboratory and clinical data of the patients according to the presence of MetS are given in Table 2.

Metabolic syndrome was diagnosed in 9 patients with recurrence of urethral stricture, and there was no statistically significant difference in terms of MetS between patients with and without recurrence (*P* = .126, likelihood ratio = 1.884). The use of postoperative 14-F CISC was found in 31.1% of patients with recurrence and 13.8% of patients with non-recurrence, and a statistically significant difference was observed in terms of increasing the risk of recurrence (*P* = .018, likelihood ratio = 5.384).

In group A, 56% of the patients had postoperative UTI, except for MetS variables, and a statistical difference was observed (*P* = .001, Pearson chi-square = 12.832).

When the patients were divided into 2 groups according to the postoperative follow-up duration with a urethral catheter as 0-3 days (n = 56) and ≥4 days (n = 76), there was no statistically significant difference between the 2 groups in terms of recurrence (*P* = .714). In addition, there was no statistically significant difference in terms of increasing the risk of postoperative UTI according to the urethral catheter indwell duration (*P* = .859).

The preoperative and postoperative parameters of uroflowmetry of patients in group 1 and group 2 are given in Table 3. Postoperative *Q*_ave_ was statistically lower in group 1 than group 2 (*P* = .001).

In group A and group B, 55% and 50% of patients, respectively, had a urethral stricture with a length of 1-2 cm, and they also had urethral stricture recurrence. The association of urethral stricture length with MetS and recurrence is shown in Table 4.

In the follow-up period, recurrence seen was in 34.1% of patients. The mean recurrence-free survival of patients was 47.94 ± 2.88 (95% CI: 42.29-53.58) months. The first-year cumulative recurrence-free survival was 92.8% ± 2.3% and third-year cumulative recurrence-free survival was 74.9% ± 4.7%. Group A and group B were compared in terms of recurrence according to the length of the urethral stricture, and there was no significant difference (25%, n = 9; 37.5%, n = 36, respectively, *P*_log_ _rank_ = .361, *P*_breslow_ = .545, *P*_tarone_ _-ware_ = .410).

**Figure 1.**
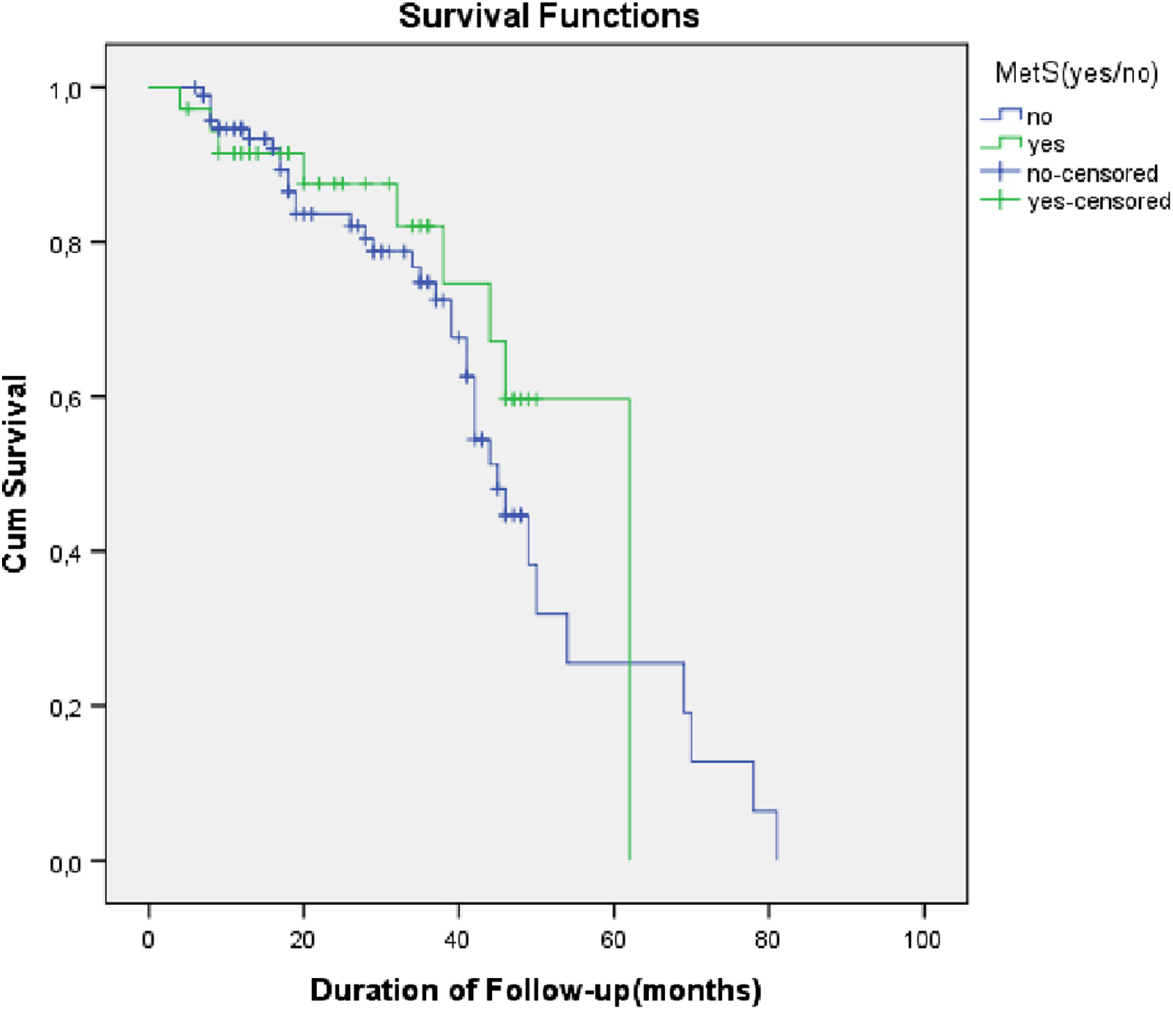
The cumulative recurrence-free survival analysis according to MetS (Kaplan–Meier analysis). MetS, metabolic syndrome.

The recurrence-free survivals were 46.44 ± 3.3 (95% CI: 40.04-52.84) months and 46.69 ± 3.8 (95% CI: 42.16-57.21) months in groups A and B, respectively (Figure 1).

The clinical characteristics of matched groups according to recurrence of urethral stricture are shown in Table 5. In matched groups according to recurrence of urethral stricture, the length of urethral stricture was between 1 and 2 cm in 2 (4.4%) patients without recurrence and in 23 (51.1%) patients with recurrence; there was a statistically significant difference (*P* = .001). The MetS in matched groups and the relationship of urethral stricture length of patients with and without MetS according to recurrence are shown in Tables 6 and 7.

The means of *Q*_ave_ and *Q*_max_ in patients with recurrence were 2.4 ± 1.4 mL/s and 4.7 ± 2.4 mL/s, respectively. The mean difference of *Q*_max_ (postoperative *Q*_max_ – *Q*_max_ in recurrence) was –8.8 ± 4.8 mL/s. The means of VV, PVR, and urethral stricture length in patients were 209.7 ± 71.9 mL, 71.9 ± 38.6 mL, and 1.5 ± 0.5 cm, respectively.

## Discussion

Metabolic syndrome predisposes to fibrosis in many tissues, and urethral stricture, which is common in the male population, may recur due to fibrosis. In our study, no relationship was observed in terms of recurrence susceptibility due to urethral stricture in patients with MetS. However, postoperative *Q*_ave_ may play an important role in predicting recurrence. Interestingly, CISC used postoperatively was found to increase the possibility of stricture.

As it is known, obesity and MetS are low-grade chronic inflammatory conditions that increase the susceptibility to fibrosis and lead to inflammatory angiogenesis.^14^ In a tissue culture study conducted by Divoux et al.^14^ it was observed that many inflammatory cytokines were secreted in adipose tissue with mast cell activation, and secondary fibrosis developed in the tissues due to this condition. In another tissue study in 2019, a positive correlation was observed between markers used in mast cell sampling in adipose tissue and immunohistochemical staining showing fibrosis such as collagen and Sirius red staining.^15^ Both studies showed that mast cells caused metabolic dysregulation through fibrosis.^14,15^ Metabolic syndrome increases reactive oxygen radicals due to hyperglycemia, creates inflammatory mediator stimulation due to macrophage activation in adipose tissue, and causes fibrosis in many tissues due to hypercholesterolemia due to dyslipidemia.^9^

The ratio of type 1 to type 3 collagen is known as 75% to 25% in normal urethral tissue. Baskin et al^1^ reported that this ratio turned to 84% to 16% in urethral stricture scar tissue. The authors concluded that changes in collagen ratios altered the structure of the urethral tissue and mainly caused stricture with decreased vascularization.^1^

The recurrence of urethral stricture in endoscopic procedures was 23%-92% and 5%-14% in open reconstructive surgical techniques.^16^ Recurrent DVIU increases the surface area of fibrosis, making it difficult to predict the incision depth.^17^ Therefore, urethral fibrosis complicates urethroplasty surgery.^18^ Repeated DVIU surgeries may be a factor predicting recurrence in patients who develop recurrence after DVIU.^19^ In recent years, DVIU is being replaced by urethroplasty. In some patients, there is a recurrent stricture after surgery due to insufficient incision in healthy tissue.^17^ In addition to surgical techniques, several factors that may trigger the recurrence of urethral stricture and increased fibrosis have been evaluated in previous studies.

In a study by Redón-Gálvez et al.^20^ 25 patients with recurrence were divided into 3 groups according to their postoperative follow-up duration with a urethral catheter as less than 7 days, 7-15 days, and more than 15 days. In these groups, recurrence occurred in 48.4%, 19.2%, and 50%, respectively, with a statistically significant difference.^20^ However, it was noteworthy that similar recurrence rates were observed in patients who had postoperative follow-up duration with a urethral catheter for less than 7 days and more than 15 days in that study.^20^ In our study, the postoperative follow-up duration with a urethral catheter made no significant difference in terms of recurrence. It was thought that postoperative recurrence was not related to urethral catheterization time.

In addition, Tian et al^21^ mentioned that severe complications could result from repeated dilations including UTI, pain, hemorrhage, hematoma, false passage, extravasation, urethral perforation, rectal injury, and sexual dysfunction in their study. The authors of the study noted that patients for whom CISC will be recommended should be selected carefully.^22^ In our study, we found a higher recurrence in the group using CISC. The authors believe that this may be due to repetitive UTIs and false passage related to patients’ education level and compliance with the CISC implementation procedure.

Considering the studies conducted according to the postoperative CISC use of patients, in the study by Kjaergaard et al^23^ in 1994, the effect of weekly CISC on the prevention of urethral stricture recurrence after internal urethrotomy was investigated in a randomized controlled trial. Recurrences were found to be significantly lower in the CISC group.^15^ In 2009, in their study with 217 patients, Lauritzen et al^24^ found that recurrence developed in 9% of the patients who used CISC and 31% of the patients who did not, and this created a statistically significant difference. In another study, in which 25 patients had recurrence, no significant difference was observed between 11 patients who underwent postsurgical periodic dilatation and 14 patients who did not.^18^

Previous investigations have proposed stricture length >1 cm as a risk factor for recurrence.^19^ The European Urology Association Guidelines recommend DVIU for primary, single, and short (below 2 cm) urethral strictures.^25^ We excluded stricture length from our analysis due to given institutional standards; commonly, DVIU is not performed for strictures >2 cm. Erickson et al^22^ found that patients with recurrence had longer strictures compared with patients without recurrence (2.29 vs. 3.5 cm; *P* < .001). In the same study, men with recurrent urethral stricture had statistically significantly lower postoperative *Q*_max_ and longer postoperative follow-up duration.^22^ In the study conducted by Mann et al^26^ in 2020, patients with stricture lengths up to 2 cm were examined and divided into 4 groups (≤0.5 cm, n = 16; 0.6-1.0 cm, n = 22; 1.1-1.5 cm, n = 9; 1.6-2.0 cm, n = 6), but it was found that there was no significant difference between the groups in terms of treatment success. In our study, the postoperative *Q*_ave_ of patients with recurrence was significantly lower, and their postoperative follow-up duration was significantly longer. There was no relationship between urethral stricture length and recurrence in our study. However, in matched pair analysis, the length of urethral stricture was significantly longer in patients with recurrence.

It is known that MetS may cause fibrosis in several tissues. However, there is no publication in the literature examining the relationship between MetS and urethral stricture. This is the first study in the literature to investigate whether recurrence of urethral stricture is more common in patients with MetS due to a susceptibility to fibrosis. Our results showed that there was no association between MetS and recurrence of urethral stricture. It was thought that this might be because MetS, which causes systemic effects, does not cause localized stricture in the urethra tissue with a paracrine effect. In addition, we found more UTIs after surgery in patients with MetS; we think this is related to MetS and its component like as DM facilitate infection.

The limitations of our study are that the study was conducted in a single center; it was retrospective, the number of patients was limited, and strictures longer than 2 cm were excluded from the study.

In this study, in which we mainly examined the relationship between MetS and urethral stricture recurrence, we found no relationship with MetS in patients with recurrence. However, a significant difference was observed in favor of patients with MetS in terms of postoperative UTIs. Despite all the findings of this study, larger randomized controlled prospective studies are needed on this subject.

## Figures and Tables

**Table 1.** Demographics and Perioperative Data of the Groups

	Group 1 (with Recurrence, n = 45) [Mean ± SD (Minimum–Maximum)]	Group 2 (Without Recurrence, n = 87) [Mean ± SD (Minimum–Maximum)]	*P* ^a^
Age (years)	49.5 ± 15.4 (20-86)	51.0 ± 19.2 (18-82)	.643
BMI (kg/m^2^)	25.9 ± 1.8 (23-32)	26.3 ± 2.4 (22-37)	.286
Time from diagnosis to surgery (months)	1.4 ± 0.7 (1-4)	1.4 ± 0.7 (1-4)	.990
Thickness of urethral catheter (*F*)	18.3 ± 1.7 (12-22)	18.8 ± 1.8 (16-22)	.136
Postoperative follow-up duration with a urethral catheter (days)	5.1 ± 2.4 (2-14)	4.5 ± 1.8 (1-7)	.130
Length of urethral stricture (cm)	1.5 ± 0.5 (0.5-2)	1.4 ± 0.5 (0.5-2)	.523
Postoperative follow-up duration (months)	33.3 ± 19.9 (4-81)	26.1 ± 13.9 (5-50)	.033^¥^

^a^Independent *t*-test.

^¥^Statistically significant.

BMI, body mass index.

**Table 2.** Laboratory and Clinical Data of the Patients According to the Presence of MetS

	Group A (with MetS, n = 36) [Mean ± SD (Minimum–Maximum)]	Group B (Without MetS, n = 96) [Mean ± SD (Minimum–Maximum)]	*P* ^a^
Fasting glucose (mg/dL)	139.0 ± 70.9 (77-463)	95.9 ± 21.3 (63-204)	.001^¥^
HDL-C (mg/dL)	38.9 ± 7.7 (26-56)	44.6 ± 8.5 (25-84)	.001^¥^
Triglyceride (mg/dL)	190.3 ± 109.2 (42-474)	131.3 ± 64.7 (57-451)	.004^¥^
Systolic blood pressure (mm Hg)	124.2 ± 9.0 (110-150)	118.9 ± 9.0 (100-140)	.004^¥^
Diastolic blood pressure (mm Hg)	81.7 ± 9.1 (60-100)	77.1 ± 9.5 (60-90)	.013^¥^
Waist circumference (cm)	96.8 ± 10.3 (80-130)	89.8 ± 6.4 (75-105)	.001^¥^
BMI (kg/m^2^)	27.2 ± 2.9 (23-37)	25.7 ± 1.7 (22-32)	.004^¥^

^a^ Independent *t*-test.

^¥^Statistically significant.

BMI, body mass index; HDL-C, high-density lipoprotein-cholesterol; MetS, metabolic syndrome.

**Table 3.** Preoperative and Postoperative Uroflowmetry Parameters of the Patients

	Group 1 (with Recurrence, n = 45) [Mean ± SD (Minimum–Maximum)]	Group 2 (Without Recurrence, n = 87) [Mean ± SD (Minimum–Maximum)]	*P* ^a^
Preoperative			
*Q*_max_	5.4 ± 2.6 (1.1-9.8)	5.6 ± 2.5 (1.3-9.9)	.721
*Q*_ave_	3.0 ± 1.7 (0.7-8.8)	3.1 ± 1.5 (0.8-7.1)	.910
VV	247.9 ± 70.6 (167-457)	249.4 ± 77.7 (153-463)	.915
PVR	60.9 ± 33.7 (0-131)	53.7 ± 28.2 (0-122)	.224
Postoperative (first month)			
*Q*_max_	13.5 ± 4.8 (9.5-31.8)	15.2 ± 4.6 (9.5-30.1)	.058
*Q*_ave_	7.3 ± 2.7 (2.4-14.8)	9.5 ± 3.2 (2.4-17.3)	.001^¥^
VV	281.6 ± 78.1 (150-472)	304.1 ± 82.5 (160-520)	.127
PVR	12.9 ± 13.1 (0-44)	13.9 ± 15.5 (0-56)	.725

^a^ Independent *t*-test.

^¥^ Statistically significant.

PVR, postvoiding residual volume; *Q*_max_, maximum urinary flow rate; *Q*_ave_, mean urinary flow rate; VV, voiding volume.

**Table 4.** Association of Urethral Stricture Length with MetS and Recurrence

			Recurrence (–)	Recurrence (+)	Total	*P* ^a^
MetS (–)	Urethral stricture length (cm)	≤1	31	18	49	.874^¥^
		>1 and ≤2	29	18	47	
	Total		60	36	96	
MetS (+)	Urethral stricture length (cm)	≤1	13	4	17	.847^¥^
		>1 and ≤2	14	5	19	
	Total		27	9	36	
Total	Urethral stricture length (cm)	≤1	44	22	66	.854^¥^
		>1 and ≤2	43	23	66	
	Total		87	45	132	

^a^ Chi-square.

^¥^Likelihood ratio.

MetS, metabolic syndrome.

**Table 5.** Clinical Characteristics of Matched Groups According to Recurrence of Urethral Stricture

	Recurrence (Mean ± SD)		*P* ^a^
	(–) (n = 45)	(+) (n = 45)	
Age (years)	48.4 ± 19.7	49.5 ± 15.4	.758
BMI (kg/m^2^)	25.8 ± 1.8	28.9 ± 1.8	.953
Time from diagnosis to surgery (months)	1.3 ± 0.7	1.4 ± 0.7	.768
Thickness of urethral catheter (*F*)	18.8 ± 1.8	18.3 ± 1.8	.199
Postoperative follow-up duration with a urethral catheter (days)	4.4 ± 1.8	5.1 ± 2.4	.113
Length of urethral stricture (cm)	0.9 ± 0.2	1.5 ± 0.6	***.001***
Postoperative follow-up duration (months)	29.0 ± 14.1	33.4 ± 19.9	.235

^a^ Independent *t*-test.

BMI, body mass index.

*P*<0.05: significant.

**Table 6.** Metabolic Syndrome in Matched Groups According to the Recurrence of Urethral Stricture

		Recurrence (n/%)		P*
		(–) (n = 45)	(+) (n = 45)	
MetS (n/%)	(–)	33/73.3	36/80.0	.619
	(+)	12/26.7	9/20.0	

^*^Fisher’s exact test.

MetS, metabolic syndrome.

**Table 7.** Relationship of Urethral Stricture Length of Patients with and Without Metabolic Syndrome According to Recurrence

		Recurrence (n/%)		P*
		(–) (n = 45)	(+) (n = 45)	
MetS (–) (n = 69)	USL <1 cm	31/68.9	18/40.0	***.001***
	USL 1-2 cm	2/4.4	18/40.0	
MetS (+) (n = 21)	USL <1 cm	12/26.7	4/8.9	***.006***
	USL 1-2 cm	0/0.0	5/11.1	

^*^Fisher’s exact test.

MetS, metabolic syndrome; USL, urethral stricture length.

*P*<0.05: significant.
